# The Prevalence of Middle Mesial Canals in the Second Mandibular Molars of an Iranian Subpopulation: A Cross-Sectional CBCT Study

**DOI:** 10.7759/cureus.51179

**Published:** 2023-12-27

**Authors:** Maryam Kuzekanani, Mousa Azami Sardoei, Laurence J Walsh

**Affiliations:** 1 Department of Endodontics, Endodontology Research Center, Kerman University of Medical Sciences and Health Services, Kerman, IRN; 2 Dentistry, School of Dentistry, the University of Queensland, Brisbane, AUS

**Keywords:** second mandibular molars, iranian, prevalence, middle mesial canal, cbct

## Abstract

Background

Missed additional canals are a common reason for the failure of root canal treatments. The prevalence of additional canals in molar teeth can vary because of ethnic differences. Hence, this study aimed to determine the prevalence and distribution of middle mesial canals (MMCs) in the second mandibular molars using cone beam computed tomography (CBCT), for an adult population from Kerman in southeast Iran.

Methodology

De-identified retrospective provided CBCT scans from three private radiology centers in Kerman were reviewed (131 patients; 58 males (44.3%), 73 females (55.7%); age range: 13 to 54 years, mean age: 33). The presence of MMCs in mandibular second molars (222 teeth; 112 left and 110 right) was recorded, along with the morphology of the distal roots in the same teeth. Frequency data were analyzed using the Chi-square test and Fisher’s exact test.

Results

The overall prevalence of MMC in the second mandibular molars was 2.3%. MMCs were found both unilaterally and bilaterally. More MMCs were found in females than males (2.7% vs. 1.7%), and on the left side (2.7% vs. 1.8%), but these differences were not statistically significant (p=1.0). There was no association between the existence of MMC in the mesial root and a second canal in the distal root of the same tooth.

Conclusion

Based on CBCT scans, the overall prevalence of MMC in mandibular second molars in this population was just below one in 44. Clinicians performing endodontic treatment on second molars should check for the presence of MMC so that this possible additional canal is not missed.

## Introduction

When performing endodontic treatment, complex root canal morphology and anatomy pose a significant challenge. Clinicians need to be aware of anatomical variations, including additional canals. This is important so that they are not missed, as that could lead to poor treatment outcomes. A range of anatomical variations occur in mandibular molars. These can be identified on conventional periapical (PA) radiographs and cone beam computed tomography (CBCT) scans [1]. Checking for the presence of such anatomical variations in molar teeth is important to achieve successful root canal treatment outcomes and reduce the likelihood of re-treatment [2]. Treatment outcomes will be compromised when one or more canals are overlooked and left untreated [3].

One of the common anatomical variations in mandibular molar teeth is the middle mesial canal (MMC), also called the mesio-central canal. Its prevalence ranges from 0.26% to 53.8% in the literature. This extra canal is located between the mesio-buccal (MB) and mesio-lingual (ML) canals and may occur in both mandibular first and second molars [4,5].

According to Pomeranz et al., MMC can be categorized into three types: independent, confluent, and fin. An independent MMC is separate from the other two canals in the mesial root and extends from its orifice to the apex of the root. A confluent MMC links with either the MB or ML canals before exiting at the apex of the mesial root. In the third type, an isthmus is present between the MB and ML canal at any point from the orifice to the apex, creating a fin configuration [6].

MMCs are difficult to detect and negotiate. Failing to find and thoroughly cleanse them leaves microorganisms inside the root canal system that may contribute to long-term failure of endodontic treatment. As with other anatomical variations, the prevalence of MMC can vary between different ethnic groups living in various geographical parts of the world [7-10]. Within one racial group, the similarity of anatomical variations reflects the fact that such features are inherited [11].

While past studies have assessed the presence of MMC in mandibular first molars, little is known regarding MMC in mandibular second molars. As well, no past studies have explored whether MMCs are associated with a second distal canal in these teeth. Hence, the present study used archived CBCT scans to determine the prevalence and distribution of MMC in mandibular second molars and their associations with an additional distal canal.

## Materials and methods

This cross-sectional observational study was approved by the institutional research ethics committee (approval number: IR.KMU.REC.1395.602; registration code: 95000359). Approval was granted from the data custodians from three private radiology centers in Kerman, Iran for access to archived CBCT scans that were taken from 2020 to 2021. The scans were all de-identified. The study aimed to find the number of MMCs in CBCT scans from adults living in the Kerman region. CBCT scans from both sides of the mandible were examined for the presence of MMCs in mandibular second molars. The morphology of the distal roots in the same teeth was also assessed.

The sample size for the study was determined by power analysis using StatMate (GraphPad Software, San Diego, CA, USA), taking the expected proportion of MMC in mandibular second molars as the reference value (0.07) and using a significance level (alpha) of 0.05 (two-tailed) and a statistical power of 95%. Based on this, the required sample size was 200 mandibular second molar teeth.

The inclusion criteria for the study were that the second mandibular permanent molars were fully erupted and had closed apices. Any scans showing orthodontic treatment, full coverage dental restorations, posts, open apices, root resorption, calcifications, pathology, or developmental disorders involving these teeth were excluded from the study.

The cone beam scans of the mandible were taken using two types of dental CBCT systems: ProMax 3D (Planmeca, Helsinki, Finland) or Pax-I 3D (Vatech, South Korea). The scan parameters used for both systems were similar: a closed-mouth and maximum intercuspation position (standard view protocol; field of view 60 × 60 × 50 mm; voxel size: 0.125 mm; exposure time: 12 seconds).

The data sets were viewed using Romexis® digital imaging software (version 2.9.2) for the Planmeca system, and OnDemand 3D digital imaging software (Dexis Dental Imaging Technologies Corporation, Quakertown, PA, USA) for the Pax-I 3D system. Axial and coronal slices (thickness: 0.05 mm) were assessed at three private radiology centers by a specially trained and calibrated dental student under the supervision of five oral and maxillofacial radiologists and an endodontist. All scans were viewed by the radiologists in their related centers to verify the student's findings, as well as by the supervising endodontist. For the second molar teeth, the presence of MMC was recorded, along with the existence of a second canal in the distal root in cases where MMC was detected in the mesial root. De-identified case data for age and gender were also collected.

Data were tabulated and then analyzed using SPSS software (IBM, USA) followed by Chi-square and Fisher’s exact tests for data frequency. The threshold level of significance was p ≤ 0.05 [12-14].

## Results

CBCT scans from a total of 131 patients were examined, which included 222 mandibular second molars. 

The results of the study are summarized in Table 1. The overall MMC prevalence among 131 patients was 2.3%, with a prevalence of 2.7% in females, and 1.7% in males. The mean age of patients with MMC was 24.5 years for females and 25 for males. MMCs were more prevalent on the left side (prevalence: 2.7%) than on the right (1.8%). Two of the three cases of MMCs were bilateral. The influence of gender was not statistically significant (Fisher's exact test, P = 1.0).

**Table 1:** Subject characteristics MMCs, middle mesial canals

	Whole sample	MMCs subgroup
Number of patients	131	3
Gender	58 males (44.3%)	1 male
	73 females (55.7%)	2 female
Mean age in years (SD)	Males 33.7 yr (11.6)	25 yr
Mean age in years (SD)	Females 32.3 yr (11.9)	24.5 yr
Side	110 right-side scans	2 cases
	112 left-side scans	3 cases

**Figure 1:**
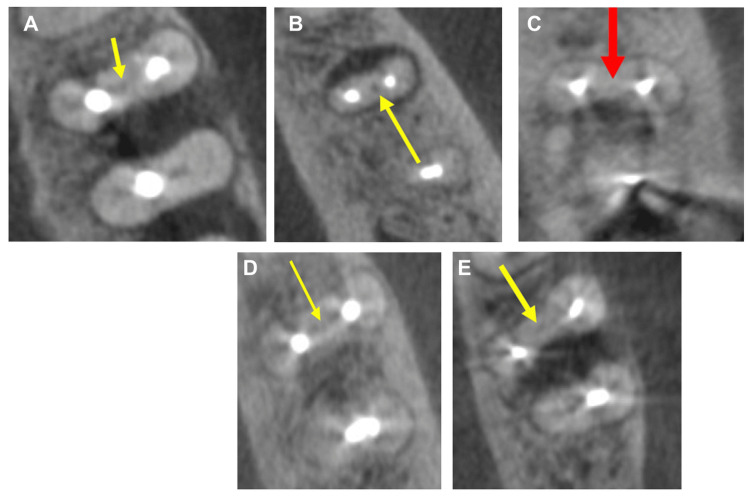
Cone beam images of MMC Panel A. Case 1. This is a right mandibular second molar (tooth 27) in a 25-year-old male, and the distal root had two canals. The left second mandibular molar in this patient was missing, so its status could not be determined. The tooth had undergone endodontic treatment, but the MMC and distal canals had not been treated. The yellow arrow points to missed MMC in the right second mandibular molar. The distal root has two canals. In this case, the left second mandibular molar had been lost. Panels B and C: Case 2. This shows bilateral MMC in a 28-year-old female, where the distal roots had only one canal on both sides. The tooth had undergone endodontic treatment, but the MMC had not been treated. Yellow and red arrows show bilateral missed MMCs, in the left and right second mandibular molars. The distal root has one canal on both sides. Panels D and E: Case 3. This shows bilateral MMC in a 21-year-old female, where both distal roots had two canals. The teeth had undergone endodontic treatment, but the MMC had not been treated. There was no notable association between the existence of MMC in the mesial root and a second distal canal in the same molar tooth. Yellow arrows show missed MMCs in the left and right second mandibular molars. The distal root has two canals on both sides. MMC, middle mesial canal

Scans from the three patients with MMC found in this study are shown in Figure 1 panels A to E. Of these, two were bilateral (cases 2 and 3). The third case (case 1) could not be assessed for symmetry as the mandibular second molar on the opposite side of the mandible had been lost.

## Discussion

Using CBCT scans, which are useful for finding additional canals in teeth, the prevalence of MMC was 2.3% in the second mandibular molars in this South Eastern Iranian population. This was higher than the rate reported as the global average for studies using CBCT scans (1.3%) (Table 2 and Table 3). The prevalence is also higher than for Saudi Arabia (0.4%). On the other hand, the prevalence is lower than in Brazil (11.1%). These differences emphasize that rates can vary due to ethnicity, a point raised in past reports [15,16].

**Table 2:** Reported rates of MMC in mandibular second molars CBCT, cone beam computed tomography; MMC, middle mesial canals

Population/country	Year and reference	Method	Prevalence
Iran - Kerman	Current study	CBCT	2.3%
Saudi Arabia	2022 [21]	CBCT	0.4%
Brazil	2022 [25]	CBCT	11.1%
All countries	2023 [27]	CBCT	1.3%
United States	2015 [24]	Guided troughing	60%

**Table 3:** Reported rates of MMC in mandibular first molars CBCT, cone beam computed tomography

Population/country	Year and reference	Method	Prevalence
Iran - Kerman	2020 [4]	CBCT	8.1%
Iran - Isfahan	2021 [22]	CBCT	3.1%
Saudi Arabia	2022 [21]	CBCT	1.3%
Pakistan	2021 [14]	CBCT	7.7%
Brazil	2022 [25]	CBCT	1.8%
Iraq (Kurdistan)	2022 [23]	CBCT	17%
All countries	2023 [27]	CBCT	4.4%
All countries	2023 [26]	Multiple methods	7%
Turkey	2016 [27]	Micro CT	14.8%
Brazil	2016 [27]	Micro CT	22.1%
United States	2015 [24]	Guided troughing	37.5%

The prevalence rate for MMC in mandibular second molars was compared to the rate in mandibular first molars for individuals living in the same location of Kerman, Iran, using data reported in a previous study, using a two-tailed Fisher’s exact test. There was no significant difference in the overall rates for unilateral or bilateral MMC (Fisher’s exact test two-tailed P = 0.30 and P = 0.25, respectively) [4]. While females were more likely to have MMC, there was no significant difference in the prevalence of MMC in females between the first and second molars (two-tailed P = 0.41). Likewise, for males, the prevalence rates for MMC were not significantly different between first and second molars (two-tailed P = 0.25). 

The frequency and distribution of different morphological variations in certain types of teeth follow inheritance patterns [15,16]. The present study showed that MMC in mandibular second molars was more prevalent in females, and this is consistent with data on MMC in mandibular first molars in the same cohort, using the same set of archived CBCT scans. No new CBCT scans were taken for either study [4]. 

Differences in the prevalence rates for MMC between studies could also result from using different methods to detect them. Available techniques include cone beam imaging, as used in the present study, conventional periapical radiographs, and guided troughing during clinical treatment [11,13].

In addition, other methods that have also been used to study the shape, morphology, and possible configurations of root canal systems of extracted molar teeth include hard tissue sectioning for histological analysis or laser scanning confocal microscopy, clearing of the teeth, and micro-CT imaging [17]. Although micro-CT may provide higher resolution and could be considered a gold standard method for assessing root canal morphology in extracted teeth, it is not practical to use in everyday clinical practice.

Cone beam imaging uses ionizing radiation and provides comprehensive data sets from which the morphology and anatomy can be assessed in three dimensions, with different possible virtual planes of sectioning. At the clinical level, cone beam imaging improves a clinician’s ability to assess root canal morphology.

The reported accuracy of cone beam imaging with a 60 × 60 mm field of view and a voxel size of 0.125 for detecting a second MB canal in maxillary molar teeth is 96% [17,18]. The cone beam scans used in the current study had the same field of view and voxel size and provided a detailed view of the morphology and anatomy of the mandibular molar roots for both of the cone beam systems that were used.

Detection of MMC in clinical studies can be undertaken using magnification devices such as operating microscopes and loupes. This approach has been used to assess the prevalence of MMC [19,20]. The rates found with these methods can be different from those with cone beam imaging (Table 2 and Table 3). 

The presence of MMC can be associated with other anatomical variations. Of the three patients with MMC in the present study, two were bilateral. In the third case, symmetry could not be assessed as the contra-lateral mandibular second molar had been lost. The finding of symmetry in most cases is consistent with the results of past studies, which have reported similar root canal morphology in paired left and right teeth in 70% of cases [20].

The present study did not find any definite association between the existence of the MMC in the mesial root and a second distal canal in the same tooth. Both single- and double-canal distal roots were seen in the mandibular second molars with the additional MMC in the mesial roots. This is in line with past findings and also a past study on first molars in the same cohort [4,21].

Several aspects of the occurrence of MMC in various populations are relevant to the present study. In a recent study using cone beam imaging to assess 395 patients from Saudi Arabia, 1377 teeth were evaluated. A total of 12 cases of MMC were found (0.9%), with nine in mandibular first molars (1.3%) and three in mandibular second molars (0.4%). There were no significant differences in terms of tooth type (first or second molar), left or right side, gender, and age (p > 0.05) [21]. The low prevalence of MMC in the first and the second mandibular molars of the Saudi population is consistent with results for the Iranian population in the present study and with past work on populations from the Middle East (Table 2) [4,14,22].

Likewise, a low prevalence of MMC was reported in a Pakistani population when cone beam imaging was used, and bilateral cases were found rarely [14]. MMC was detected in 7.7% of these teeth, and 11.4% of these had adjacent C-shaped second mandibular molars. These findings are in line with our previous study of mandibular first molars [4]. The likelihood of an adjacent C-shaped mandibular second molar is increased by three times when an MMC is present (P = 0.048, OR: 3.108) in the first mandibular molars [14].

Similarly, a cone beam imaging study of MMC in mandibular first molars in an Iranian population reported an overall prevalence of 3.13% (2.35% in females and 3.92% in males), with no significant differences between the genders (P = 0.19) [22]. On the other hand, a cone beam imaging study using a Kurdish-Iraqi population found MMCs in 14.7% of mandibular first molar teeth on the right and 19.3% on the left side. There were no significant influences of gender or age [23]. This reinforces the overall point of the present study that ethnic factors cause variations in the prevalence of MMC.

In the present study, MMCs were not instrumented in root-filled teeth. This reinforces the need to check all mandibular molar cases carefully, both radiographically and clinically, for MMC, using all methods available. Cone beam imaging can be undertaken before commencing treatment. Once endodontic treatment has started, an inspection can be undertaken using magnifying loupes and operating microscopes. A clinical study conducted in the United States used a guided troughing method under high magnification to identify very small root canal orifices. Using this approach, they found a high prevalence of MMC in mandibular molars, namely 37.5% in first molars, and 60% in second molars [24].

These findings of the current study are consistent with the results of previous studies of mandibular first molars in the Kerman region of Iran [4]. Adding to this, a recent study found that of 216 mandibular first molars, 11.1% had MMC, while for 228 mandibular second molars, only 1.75% had MMC. The presence of the MMC was significantly higher in mandibular first molars (p < 0.0001). Also, the buccolingual diameter and the distance between MB and ML canals were higher in teeth that had MMC (p = 0.024 and p = 0.005) [25]. 

A global systematic review and meta-analysis study published in 2023 reported an overall incidence of MMC ranging from 1% to 23% in mandibular first molars worldwide, based on the regions. They estimated a global prevalence of around 7% [26,27]. The rate for the first mandibular molars reported in 2020 in an Iranian cohort was 8.1%, which is similar to the global average rate (Table 3). No significant influence or association was reported for gender, left-right side, and age for MMC [26].

A 2016 study assessed extracted mandibular first molars collected from Brazilian and Turkish populations, using micro-CT with a high resolution (voxel size: 9.9 μm). That study found a prevalence of 22.1% for the Brazilian population and 14.8% for the Turkish population [11]. Once again, these results reinforce that the prevalence of MMC varies according to the ethnic background of the population being studied.

Another global systematic review and meta-analysis study published in 2023 reported that MMC occurs more often in mandibular first molars than in mandibular second mandibular molars. That study also confirmed the previously mentioned point that the prevalence of MMC is not influenced by age, gender, and the side of the mouth [27].

Overall, the variations seen for MMC prevalence values in the literature reflect both genetic factors (ethnic background), as well as the sensitivity of the detection method used, and the criteria applied for reporting this anomaly (an isthmus or an independent canal). These influences explain the variable prevalence values for MMC in mandibular first and second molars in different geographical parts of the world, as summarized in Table 2 and Table 3. Further research is recommended using sensitive methods to better map the prevalence and distribution of MMC in various populations. worldwide.

## Conclusions

Based on cone beam scans, the overall prevalence of MMCs in mandibular second molars of the Kerman population in this study was 2.3%. MMCs were found more often in females and can be bilateral. There was no association of MMC in the mesial root with a second canal in the distal root. Clinicians who are performing endodontic treatment on mandibular second molars should check carefully to ensure that MMC has not been overlooked. Techniques such as inspection using magnifying loupes or dental microscopes and ultrasonic guided troughing can be useful for this purpose.
